# A Medical Causerie

**Published:** 1916-02-26

**Authors:** 


### A MEDICAL CAUSERIE.

Some of the newspapers recently expressed great concern at the rumour that the War Office had decided to withdraw all sick and wounded soldiers from the civil hospitals and to treat all such patients at military hospitals only. To judge from the comments, the conclusion might have been formed that such a step had inflicted grave injustice on the civil institutions and had invaded their legitimate duty and function. It never seems to have dawned on the critics that except in institutions where additional and special accommodation had been provided every bed occupied in a civil hospital by a sick or wounded soldier was a bed subtracted from the service of the ordinary population. The amount of suffering—to mention no more serious consequences—which has resulted from this arrangement has been very great. For months it has been necessary to refuse applicants who in ordinary circumstances would have been admitted as urgent cases, and both these and the more chronic illnesses have had to put up with the scanty help and opportunities of their homes. The medical and surgical staffs of the hospitals are well aware of these facts, and, while more than willing to help the soldiers, have regretted the sufferings inflicted on the rejected patients. Hence the news that the military authorities were able to provide for the soldiers without prejudicing the opportunities of civilians ought to have caused satisfaction rather than regret. It, of course, meant that the military hospitals were proving equal to the duties for which they were created, and also that the civil hospitals were set free for their special and proper purposes. Soldiers and civilians alike merit consideration, and an arrangement which showed that both could receive proper attention ought to have been welcomed as good news, instead of being made a matter of reproach. It is a one-sided variety of patriotism which cannot rejoice that there is hospital accommodation for all.

The hostility recently shown towards the Central War Committee has a really substantial basis, though it has not always been expressed in the most seemly fashion. One ground undoubtedly is that interests which have a right to be heard are not adequately represented, and another that many men who are able and willing to give part-time service are not being utilised. The former may not easily be removed, seeing that the constitution of the committee was definitely fixed at the representative meeting of the British Medical Association at which it was appointed. Yet it would certainly be well if some of the interests more immediately concerned were asked to send representatives to the committee, and in times like the present rules and regulations must not be allowed to strangle efficiency. For those who insist upon the letter of the law it is always possible to go to the next representative meeting and to ask for a vote of indemnity for any technical breach of the former instruction. There certainly was a desire, at least in some quarters, to keep the working of the scheme in the hands of the British Medical Association, but circumstances have forced to the front far wider considerations, and with this new development it is necessary to reckon. It was perhaps unfortunate that both the chairman and one of the honorary secretaries should be official members of the Association, and the latter, one would have thought, had already sufficient to occupy his attention. Indeed, one source of dissatisfaction is the restriction of responsibility to a comparatively small number of men, who, like a stage army, appear again and again though in different capacities. There are plenty of men who are not less capable and are quite willing to help, but the powers that be do not seem able to look beyond the narrow circle of their own ranks.

With regard to the complaint that many men who have taken commissions in the R.A.M.C. have little to do, it is a perfectly sound answer to say that the authorities must have at their disposal a sufficient number of men to meet the maximum demand, and that this necessarily means a number in excess of what is required when the fighting is on a limited scale. But to claim that the medical officers at present serving in France all find themselves fully occupied and that everything is for the best in this best of all possible worlds is to spoil a good case by overstating it. A gentleman who has paid a flying visit to some of the hospitals and has evidently had a pleasant time, may write to the Press in this *couleur-de-rose* strain, but his enthusiasm will only excite a smile in those who are behind the scenes. In war-time it is something that everyone has made the best of circumstances, and those who find that nothing but the pink of perfection can be seen will be suspected of wearing spectacles which are so strongly tinted that they permit only the rays which enjoy an official sanction.

The war has brought a new responsibility to certain medical certificates, as the authorities are now very strict in reference to the granting of passports even for those who are medically advised to go abroad for the benefit of their health. Unless the medical opinion is that such a course is absolutely essential the application is likely to be refused. Mere advisability is not sufficient, and it may be difficult for a doctor to rise to imperative terms even when he has no doubt of the wisdom of the course he recommends. This may mean hardship for individuals, but the responsibility rests not with the practitioner but with the Foreign Office, and in the circumstances it cannot be a matter for surprise that the authorities are strict. Not all the discoveries and surprises reach the public ear, but sufficient is known to make it certain that spies are busy in various directions and that they adopt many ingenious and plausible pretexts to gain their purpose. It is necessary to be careful that medical certificates, given with the best intention and in all good faith, are not utilised for treacherous ends.

